# Supplementary material for: Pyruvate kinase type M2 promotes tumour cell exosome release via phosphorylating synaptosome-associated protein 23
Source: Nat Commun. 2017 Jan 9;8:14041. doi: 10.1038/ncomms14041 (PMC5228053; doi:10.1038/ncomms14041)
Supplement: Supplementary Information — Supplementary Figures, Supplementary Table. [file ncomms14041-s1.pdf]

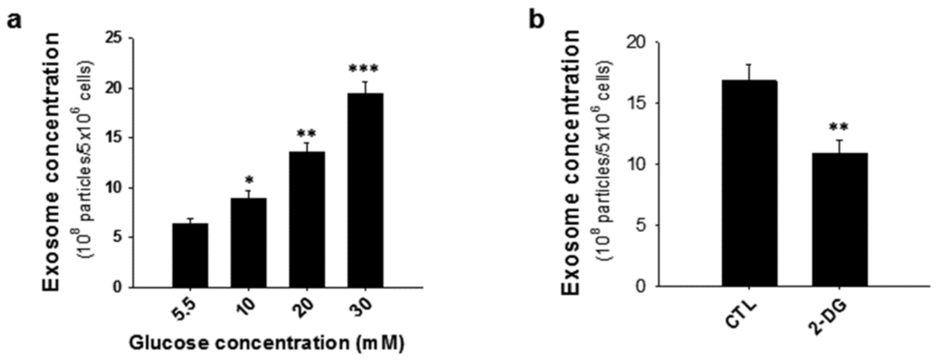

Supplementary Figure 1. Effect of cellular glycolysis on tumor cell exosome secretion. A549 cells were cultured in medium containing different concentration of glucose (**a**) or treated the inhibitor of glycolysis 2-DG (10 mM) (**b**). *P <0.05, **P <0.01, *** P <0.001 as determined by the one-way ANOVA test (**a**) and *t*-test (**b**).

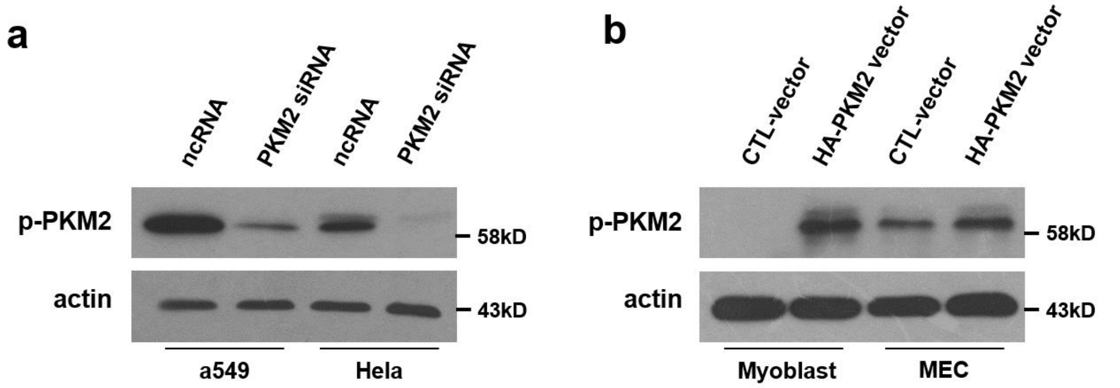

Supplementary Figure 2. Phosphorylated PKM2 (p-PKM2) levels in tumor or non-tumor cells. **(a)** Western blot detection of p-PKM2 in A549 and Hela cells after PKM2 knockdown. **(b)** Western blot detection of p-PKM2 in myoblast and MEC cells after PKM2 overexpression.

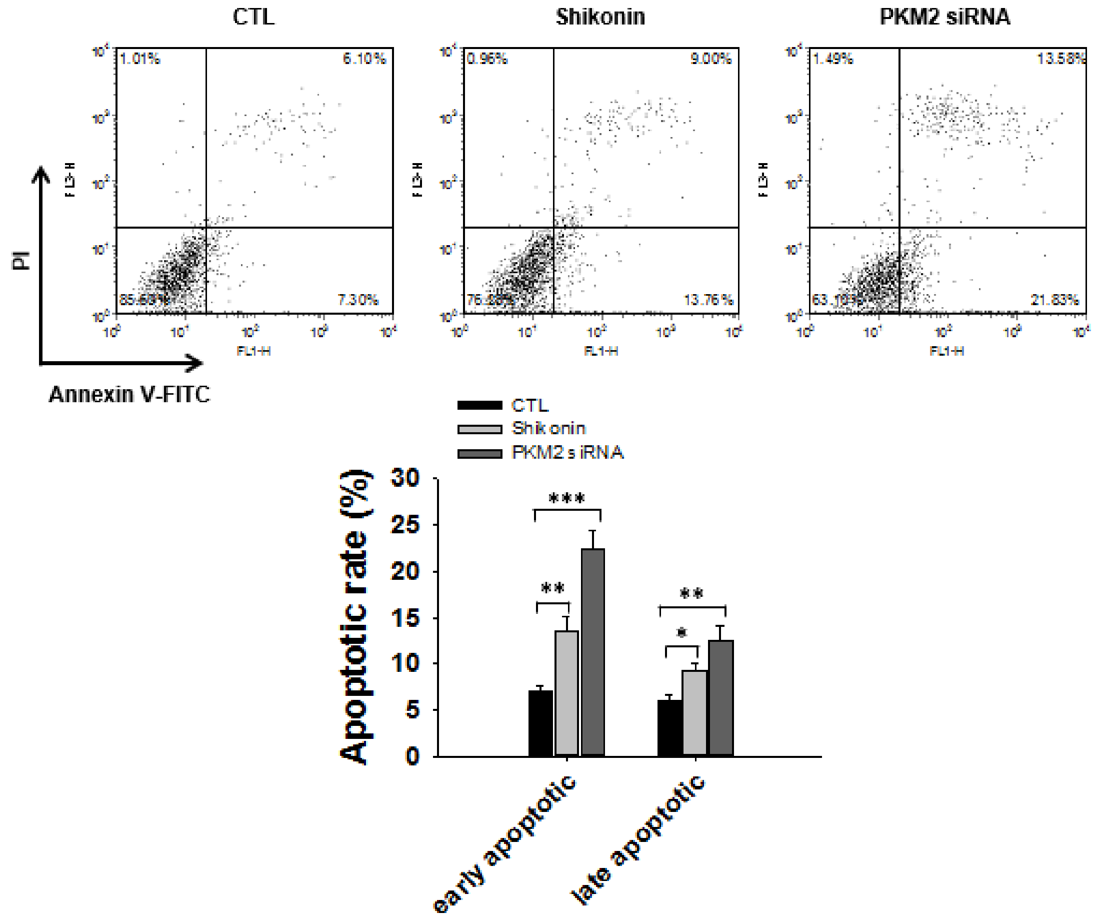

Supplementary Figure 3. A549 cell apoptosis detected by flow cytometry after glycolysis inhibitor shikonin treatment or PKM2 knockdown. **P* <0.05, ***P* <0.01, *** *P* <0.001 as determined by the one-way ANOVA test.

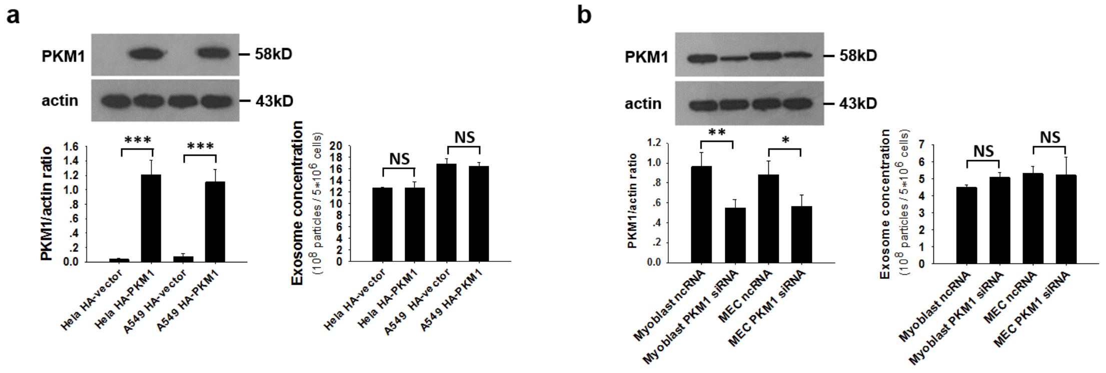

Supplementary Figure 4. Pyruvate kinase activity of PKM is not relevant to tumor cell exocytosis. (**a**) Overexpression of PKM1 in Hela and A549 cells, which display low level of PKM1, has no effect on the release of exosomes. Left, cellular PKM1 level assessed by western blot analysis. Right, concentration of exosomes detected by NTA. (**b**) Decrease of PKM1 level in primary myoblasts and MECs, which have high level of PKM1, does not affect cell exocytosis. Left, cellular PKM1 level assessed by western blot analysis. Right, concentration of exosomes detected by NTA. *P <0.05. **P <0.01. ***P <0.01 as determined by t-test.

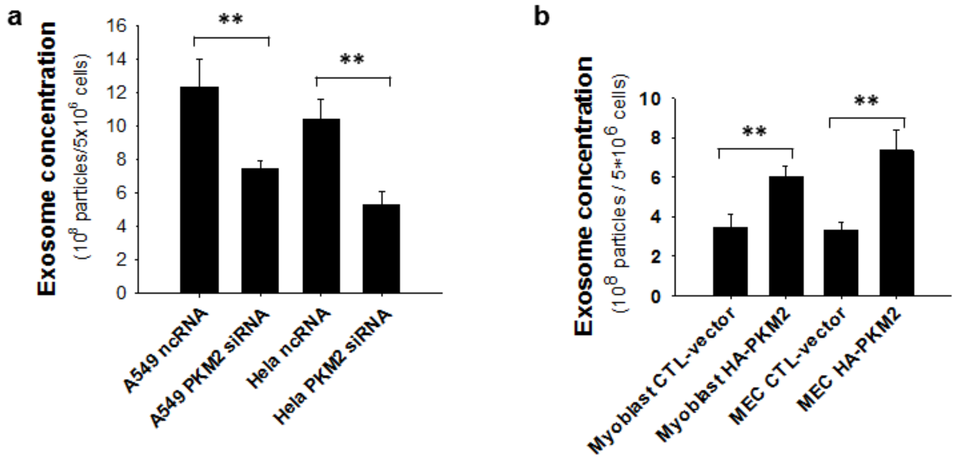

Supplementary Figure 5. NTA detection of exosome release from tumor or non-tumor cells. Exosomes were isolated from A549 and Hela cells after PKM2 knockdown **(a)** or from myoblast and MEC after PKM2 overexpression **(b)** using sequential centrifugation methods (see Method section). **P* <0.05, ***P* <0.01 as determined by *t*-test.

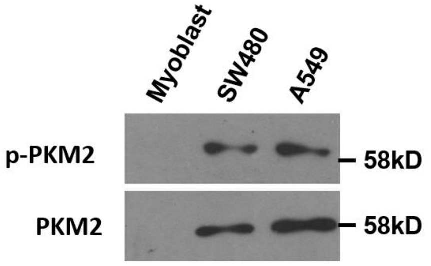

Supplementary Figure 6. Western blot detection of phosphorylated PKM2 in exosomes secreted from myoblast, SW480 or A549 cells.

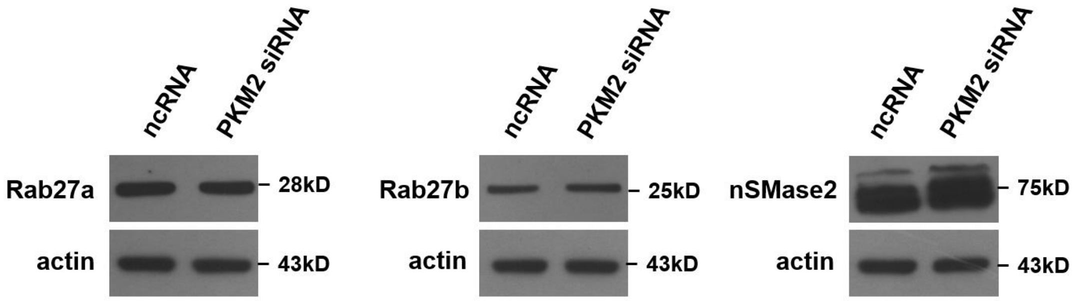

Supplementary Figure 7. Western blot detection of Rab27a, Rab27b and nSMase2 levels after PKM2 knockdown.

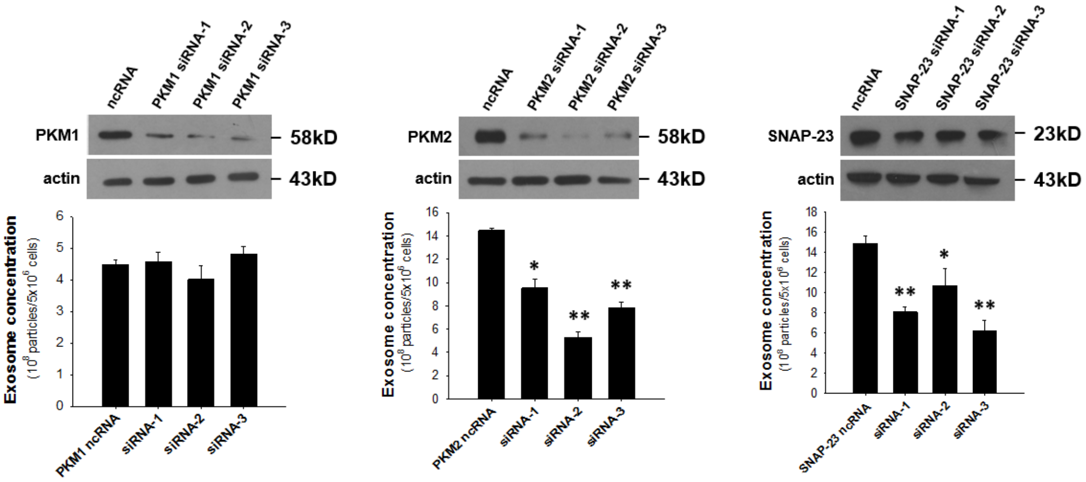

Supplementary Figure 8. Western blot detection of the knockdown effects of PKM1, PKM2 and SNAP23 siRNA oligos. NTA assays showed the similar phenotype of 3 siRNA oligos against one given target. **P* <0.05. ***P* <0.01 as determined by the one-way ANOVA test.

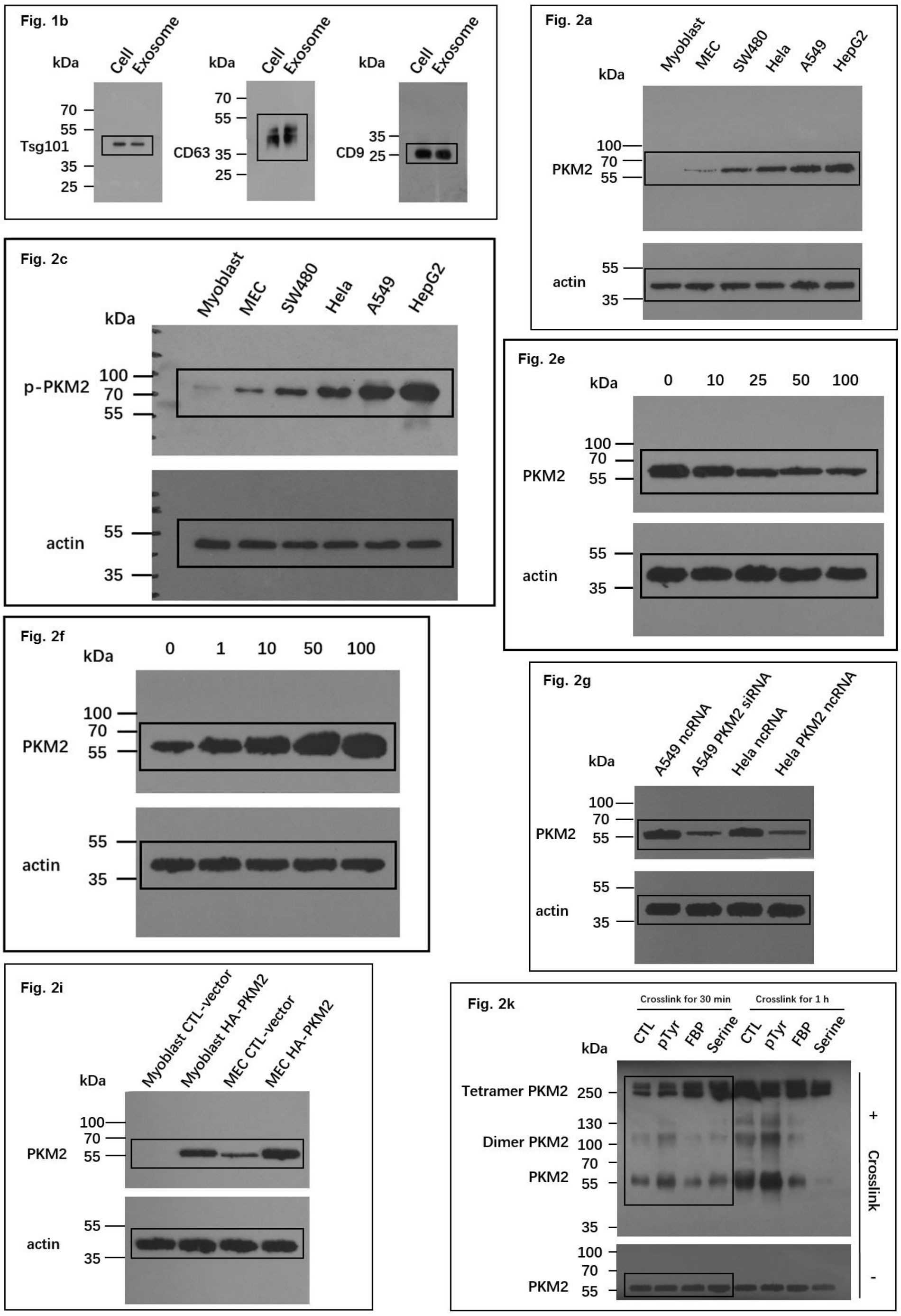

Supplementary Figure 9a. Uncropped data. The uncropped illustrations used to prepare the main and supplementary figures of this manuscript are shown with reference to their specific sub-figures indicated on each panel.

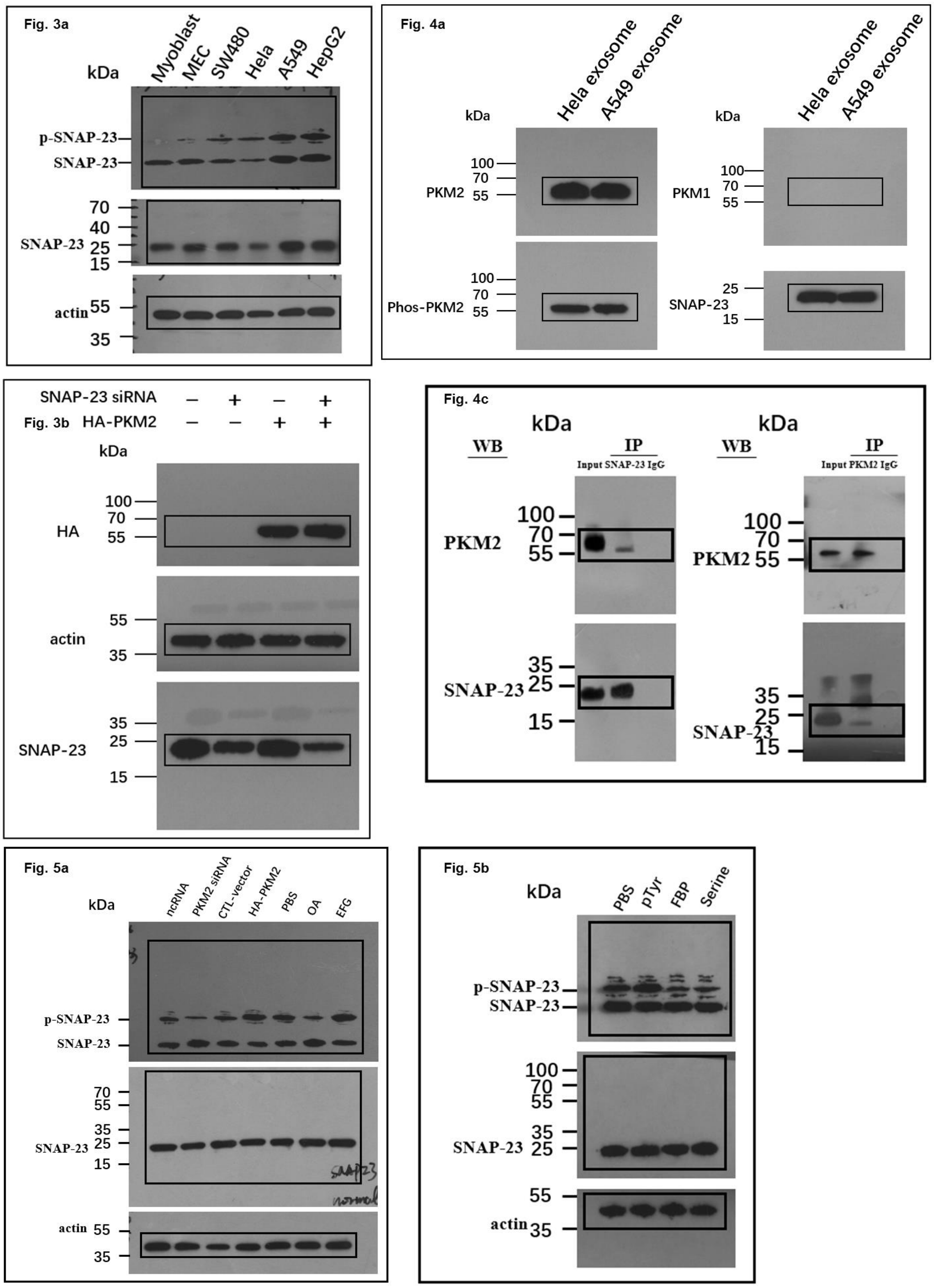

Supplementary Figure 9b. Uncropped data. The uncropped illustrations used to prepare the main and supplementary figures of this manuscript are shown with reference to their specific sub-figures indicated on each panel.

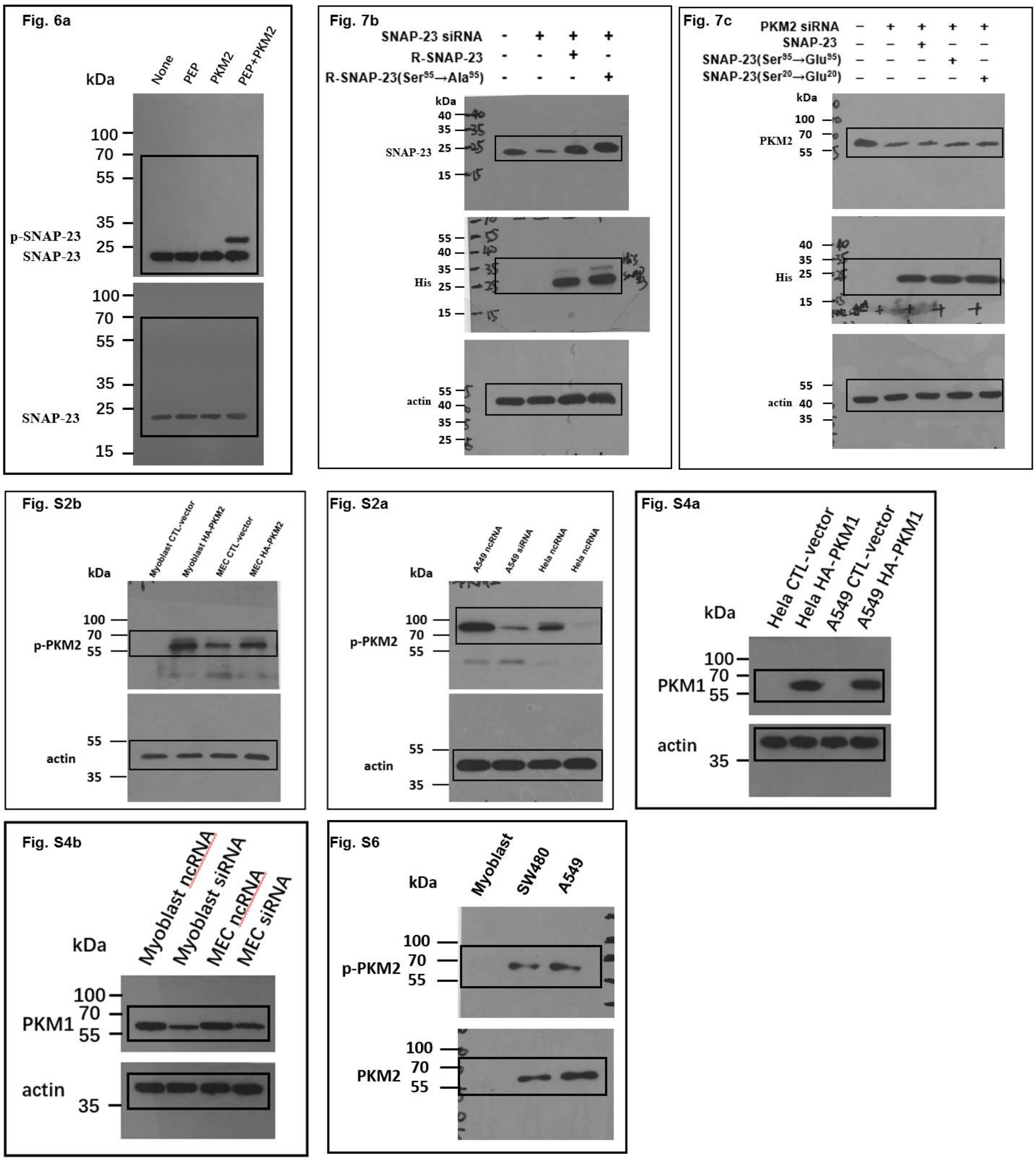

Supplementary Figure 9c. Uncropped data. The uncropped illustrations used to prepare the main and supplementary figures of this manuscript are shown with reference to their specific sub-figures indicated on each panel.

Supplementary Table 1. Proteins detected by iTRAQ and MS assay		
Accession number	***Description***	
O00161	HUMAN Synaptosomal-associated protein 23	(5)
P14618	Pyruvate kinase PKM2	(4)
Q15836	Vesicle-associated membrane protein 3	(2)
P51809	Vesicle-associated membrane protein 7	(2)
P62820	Ras-related protein Rab-1A	(2)
P61019	Ras-related protein Rab-2A	(2)
P20339	Ras-related protein Rab-5A	(2)
P61020	Ras-related protein Rab-5B	(2)
P51148	Ras-related protein Rab-5C	(2)
P51151	Ras-related protein Rab-9A	(2)
P61106	Ras-related protein Rab-14	(2)
Q13636	Ras-related protein Rab-31	(2)
Q9Y3P9	Rab GTPase-activating protein 1	(2)
Q9UNE2	HUMAN Rab effector Noc2	(2)
O95295	SNARE-associated protein Snapin	(1)
P63027	Vesicle-associated membrane protein 2	(1)
Q9UEU0	Vesicle transport through interaction with t-SNAREs homolog 1B	(1)
Q96S21	Ras-related protein Rab-40C	(1)

Supplementary Table 1. Detection of PKM2 but not PKM1 in exosome fraction derived from SW480, A549, Hela, 293T and LLC cells by iTRAQ and MS assay. The number in the parenthesis stands for the times that the protein has been detected in exosome fraction released by 5 different cell lines. As shown in the table, SNAP-23 is the only SNARE family protein detected in exosomes derived from all the 5 cell lines. Some proteins of exocytosis-related Rab protein family are also in the list.
